# Naturalist Identity and Biodiversity Conservation: The Mediating Role of Obligation

**DOI:** 10.1002/pchj.70098

**Published:** 2026-05-04

**Authors:** Yichuan Meng, Jin Chen

**Affiliations:** ^1^ Laboratory of Tropical Forest Ecology, Xishuangbanna Tropical Botanical Garden Chinese Academy of Sciences Menglun, Mengla China; ^2^ University of Chinese Academy of Sciences Beijing China

**Keywords:** biodiversity conservation action, factor analysis, identity theory, mixed methods, naturalist identity scale, norm activation model

## Abstract

Identity is a fundamental concept in social psychology; however, its application in conservation education has been limited. This study examines the impact of naturalist identity on biodiversity conservation behaviors, focusing on both direct and indirect pathways mediated by a sense of obligation. Using a mixed‐methods approach, we developed and validated a 10‐item Naturalist Identity Scale (NIS) using a total of 824 valid responses from naturalists and college students, demonstrating strong psychometric properties and cross‐group comparability. Results from 198 naturalists revealed that naturalist identity significantly predicts biodiversity‐related actions, with a sense of obligation partially mediating this relationship. Consequently, the study provides a reliable scale for measuring naturalist identity and suggests that a stronger naturalist identity is associated with a higher frequency of biodiversity actions. Our findings emphasize the importance of identity in fostering conservation behaviors and offer practical insights for environmental education strategies.

## 1 Introduction

Global biodiversity is currently facing a crisis, with human activities accelerating the extinction of species and the degradation of ecosystems. Consequently, the preservation of biodiversity has become an imperative task for contemporary society (Davis et al. 2018; Johnson et al. 2017). Effective conservation strategies depend not only on policy interventions and scientific advancements but also on the active engagement of individuals and communities in conservation efforts (Crimston et al. 2016; Lengieza and Swim 2021; Stern et al. 1999). A fundamental aspect of these initiatives is the psychological dimension of conservation, particularly how individuals perceive their relationship with nature and their moral responsibilities toward its protection (Ghasemi and Kyle 2021; Richardson et al. 2020). The field of social psychology in biodiversity conservation explores how psychological factors, such as identity and values, influence conservation behaviors, thereby offering significant insights for education and policy development (Mastrangelo et al. 2014; Stern et al. 1999; Wauters et al. 2016).

Naturalists represent a distinctive group within the broader community engaged in biodiversity conservation and often play a crucial role in these efforts. Characterized by their intrinsic curiosity and passion for nature, naturalists not only participate in scientific exploration but also act as stewards of biodiversity (Merenlender et al. 2016; Robbins et al. 2021). Historically, the role of naturalist has been associated with amateur practices, including specimen collection and field observation (Miller‐Rushing et al. 2012). In recent decades, there has been a global resurgence in amateur naturalism, particularly through activities such as birdwatching (Dillon et al. 2016; Merenlender et al. 2016). In China, a notable increase in naturalist programs, including birdwatching and biodiversity clubs, reflects a growing interest in natural history and conservation, especially among the younger individuals (China Wildlife Conservation Association 2024). Naturalists often transcend the role of mere observers; their profound connection to nature and expertise in biodiversity position them as key contributors to community‐based conservation initiatives. E. O. Wilson, a distinguished biologist and naturalist, underscored the significance of popularizing the role of naturalists in society, asserting that the promotion of amateur naturalists can enhance the public's sense of connection to nature—an essential element for inspiring conservation initiatives and addressing environmental challenges (Kellert and Wilson 1993). As the global population of naturalists expands, several critical questions emerge: How might this trend influence broader conservation efforts? To what extent does a personal passion for nature translate into measurable conservation outcomes? Furthermore, what potential challenges or limitations exist in relying on naturalists as pivotal actors in biodiversity preservation? Addressing these questions will enhance our understanding of the complexities surrounding the role of naturalists in conservation, thereby necessitating further empirical research.

### 1.1 Theoretical Background

#### 1.1.1 Naturalist Identity

The concept of identity has garnered significant attention in social psychology, particularly in relation to behavior and group membership (Pronin 2008; Stryker and Burke 2000). Recent studies have begun to explore how identity—specifically environmental identity—shapes proenvironmental behaviors. Studies indicate that individuals who strongly identify with the environment are more likely to engage in behaviors aimed at its protection (Freed 2018; Shang and Wu 2022). While much of the existing literature has focused on environmental identity, there has been comparatively less exploration of naturalist identity. Few studies have specifically examined this aspect, with notable contributions including the work of Hecht et al. (2019), who explored the development of interest in nature across various learning environments, illustrating how individuals evolve into naturalists throughout different stages of their learning experiences. Similarly, Hecht and Nelson (2021) investigated the impact of environmental education practices on the development of participants' naturalist identities. Their findings underscore the significance of relational processes and naturalist practices in shaping young people's connection to nature and their developing identities as naturalists. However, existing research on naturalist identity has primarily been qualitative, with the only quantitative assessment limited to a 2‐item self‐report scale (Meng et al. 2025), leading to a scarcity of quantitative studies that rigorously examine this construct.

Other efforts to develop identity scales have included hobbyist groups. Several multidimensional frameworks have been proposed, typically comprising three (recognition, competence, and performance), four (interest, recognition, competence, and performance), or five dimensions (identity variable, interest, recognition, competence, and performance) (Carlone and Johnson 2007; Hazari et al. 2010; Hosbein and Barbera 2020). Early identity models primarily focused on three dimensions—identity, competence, and performance—arguing that these dimensions collectively represent a group's identity. However, these scales may be more applicable to developed hobbyists rather than beginners. Some studies have also introduced the interest dimension (Hazari et al. 2010), specifically designed for those individuals who have an interest in nature but lack competence. These dimensions are particularly relevant for assessing naturalist identity, offering a more inclusive framework for both experienced individuals and novices.

#### 1.1.2 Nature Connectedness, Nature Exposure and Nature Experience

Recent research has highlighted the crucial roles of nature connectedness, nature exposure, and nature experience in fostering proenvironmental behaviors and the development of a naturalist identity. Nature connectedness is defined as the emotional, cognitive, and behavioral relationship individuals have with the natural world (Schultz 2002). Studies show that individuals with higher nature connectedness are more likely to engage in environmentally responsible actions (Barrera‐Hernández et al. 2020; Richardson et al. 2020). This sense of connection enhances proenvironmental attitudes, which can translate into tangible conservation actions. Nature exposure, characterized by the amount of time spent in natural environments, is pivotal in strengthening nature connectedness. Research indicates that increased nature exposure fosters a greater emotional attachment to nature and encourages sustainable behaviors (Alcock et al. 2020; Martin et al. 2020). Furthermore, nature experience, which includes direct and active engagements with nature (e.g., hiking or birdwatching), deepens individuals' connection to the environment. These experiences not only enhance emotional bonds with nature but also reinforce one's naturalist identity (Meng et al. 2025; Straka et al. 2025). Even brief interactions with nature have been shown to elicit positive environmental actions (Flecke et al. 2024). Collectively, these constructs provide a framework for understanding how individuals engage with nature, develop emotional connections to it, and cultivate their naturalist identities, which may subsequently motivate them to participate in conservation efforts.

#### 1.1.3 Naturalist Identity, Sense of Obligation, and Biodiversity Actions

Previous research has established a connection between environmental identity and biodiversity conservation (Wesolek 2020); however, there is a paucity of studies specifically examining the role of naturalist identity in fostering biodiversity conservation actions. The Norm Activation Model (Schwartz 1977) posits that when individuals perceive environmental threats as morally significant, they develop a sense of obligation to act, which subsequently motivates them to engage with environmental issues. This sense of moral responsibility serves as a crucial mediator between identity and behavior. Van Der Werff et al. (2013) demonstrated that environmental identity frequently elicits feelings of obligation. Recent studies, such as those conducted by Janmaimool and Khajohnmanee (2020), have indicated that this sense of obligation is a strong predictor of proenvironmental behaviors. Nevertheless, the specific influence of naturalist identity on biodiversity conservation actions through this sense of obligation remains insufficiently explored, particularly in non‐Western contexts, such as China.

### 1.2 Research Framework

This study is grounded on two key theoretical frameworks: Identity Theory and the Norm Activation Model, both of which are essential for understanding conservation behavior. Identity theory, as proposed by Stryker and Burke (2000), asserts that individuals' behaviors are influenced by their identification with specific social groups, which in turn affects their actions and decision‐making processes. In the context of naturalists, this theory suggests that individuals who identify as naturalists are more inclined to engage in behaviors that are consistent with this identity, such as participating in biodiversity conservation initiatives. Furthermore, a strong identification with this role enhances intrinsic motivation, driven by a sense of moral obligation, thereby encouraging individuals to take proactive measures for conservation (Van Der Werff et al. 2013). This psychological perspective highlights how identity not only shapes an individual's relationship with nature but also motivates behavior through a sense of moral responsibility. The Norm Activation Model, developed by Schwartz (1977), offers a complementary perspective by positing that personal norms, which are activated by feelings of moral obligation, play a crucial role in motivating proenvironmental behavior. This integrated framework underscores the mediating function of a sense of obligation in translating identity into action, making it a vital variable for understanding how naturalist identity influences actions related to biodiversity conservation.

### 1.3 Research Aims and Hypotheses

To address the identified gaps in the literature, we designed a cohesive series of three studies. First, we aimed to develop and validate a Naturalist Identity Scale (NIS) that accurately reflects the multidimensional nature of the construct. Second, we evaluated the psychometric properties and cross‐group applicability of the NIS among both naturalists and college students. Third, we investigated the predictive role of naturalist identity in relation to biodiversity conservation actions, specifically testing whether this relationship is mediated by a sense of obligation. The following hypotheses were proposed:

*Naturalist identity positively predicts actions related to biodiversity conservation*.

*Naturalist identity positively predicts individuals' sense of obligation toward conservation actions*.

*A sense of obligation mediates the relationship between naturalist identity and conservation actions*.

## 2 Study 1

### 2.1 Sample and Data Collection

#### 2.1.1 Interview

This study was conducted at the Xishuangbanna Tropical Botanical Garden (XTBG), which is actively involved in natural history education and research, attracting a diverse group of naturalists. We conducted face‐to‐face interviews with 10 selected naturalists, following Hecht et al.'s (2019) classification. These individuals specialized in various fields, including spiders, plants, insects, astronomy, and geology (Appendix A). To develop a framework for naturalist identity, we referenced identity frameworks from physical science and scientific identity (Hazari et al. 2010; Hosbein and Barbera 2020), encompassing four dimensions: interest, identification, competence, and performance. Based on this framework, we created an eight‐question interview outline in a funnel format (Appendix A), with questions such as, “As a naturalist, what specific areas of interest do you pursue?” Interviews were conducted in September 2023, with eight participants interviewed in person and others via voice calls. With participants' consent, interviews were recorded, lasting between 16 and 76 min.

#### 2.1.2 Factor Analysis of the NIS

We distributed the questionnaire by sharing Quick Response codes in large WeChat and Tencent‐QQ groups associated with naturalists, including the “China Insect Taxonomy Group,” “Wuhan Birdwatching Group,” and “CHF‐Naturalists Group.” An informed consent form was provided at the beginning of the questionnaire, and participants were informed about the study's purpose. They had the option to voluntarily complete the questionnaire or decline participation. We used Questionnaire Star to develop an electronic version of the questionnaire. In addition to the original NIS, the questionnaire gathered demographic information, including the participant's gender, age, and education level (Appendix B). The electronic questionnaire was distributed to various naturalist groups in January 2024. A total of 436 questionnaires were collected from the naturalist groups. Following the exclusion of 20 invalid responses due to inconsistent answers on the reverse‐scored items and excessively brief completion times, we retained 416 valid questionnaires (Table 1), resulting in an effective response rate of 95.4%.

**TABLE 1:** The demographic information of the sample used for factor analysis in Study 1 (*N* = 416).

Variables	Number of participants	Percentage (%)
Gender		
Male	174	41.8
Female	242	58.2
Age		
< 20	37	8.9
20–29	163	39.2
30–39	99	23.8
40–49	71	17.1
≥ 50	46	11.1
Education		
Middle school	17	4.1
High school	27	6.5
Undergraduate degree	243	58.4
Postgraduate degree	129	31.0

*Note:* Percentages may not total 100% exactly due to rounding.

### 2.2 Data Analysis

#### 2.2.1 Interview

We employed a general inductive approach to analyze the interview data, which provides a systematic, straightforward method for qualitative analysis without requiring a deep understanding of a specific qualitative framework (Thomas 2006). Audio recordings of the interviews were transcribed using iFLYTEK software, and the resulting transcripts were coded with MAXQDA (2022) software. This approach, combined with a literature review, enabled the development of preliminary items for the NIS. Items mentioned by more than two participants were selected, leading to 18 potential scale items. Additionally, by reviewing existing identity scales in the fields of physics, science, chemistry, and environmental studies (Cheung et al. 2016; Hazari et al. 2010; Hosbein and Barbera 2020; Walton and Jones 2018), we generated 7 additional items, resulting in a preliminary pool of 25 items. These items were then reviewed by a panel of 12 experts in scale design and environmental education, each with expertise in natural history. Experts evaluated the suitability of each item for inclusion, providing feedback and recommendations.

#### 2.2.2 Factor Analysis of the NIS

The reliability and validity of the five‐point Likert scale were assessed through item selection and various statistical analyses. Discrimination coefficient analysis evaluated each item's ability to distinguish between different levels of the measured construct, while correlation analysis examined item coherence. Internal consistency was confirmed using Cronbach's alpha (*α*) and construct reliability (CR). For dimensional structure validation, a cross‐validation approach was employed, integrating exploratory factor analysis (EFA) and confirmatory factor analysis (CFA). EFA identified potential factor structures, and CFA tested the model fit, ensuring stability and generalizability (Brown 2015; Hurley et al. 1997). Fit indices such as the Chi‐square to degrees of freedom ratio (*χ* ^2^/df), root mean square error of approximation (RMSEA), and comparative fit index (CFI) were used to assess model fit, balancing parsimony and explanatory power (Hu and Bentler 1999). Validity was further evaluated through content validity, criterion‐related validity, convergent validity, and discriminant validity. For CFA‐based item and factor retention, convergent validity was evaluated using average variance extracted (AVE) and construct reliability (CR). Factors with inadequate convergent validity (e.g., AVE < 0.50) were considered for removal or further revision, even if they initially emerged in the EFA, because the final scale structure was determined based on both exploratory results and confirmatory evidence. Data analysis was performed using the R packages “psych” and “lavaan.”

### 2.3 Results

#### 2.3.1 Original Version of NIS

The version of the NIS developed by the expert panel comprises 20 items (Appendix C). This scale includes 4 items related to the interest subdimension, 4 items pertaining to the recognition subdimension, 6 items associated with the competence subdimension, and 6 items corresponding to the performance subdimension.

#### 2.3.2 Exploratory Factor Analysis

Prior to conducting the EFA, we assessed item homogeneity and discriminative power. The corrected item‐total correlations (CITC) ranged from 0.5 to 0.7, with an overall Cronbach's *α* exceeding 0.9, indicating strong internal consistency. Removal of any item did not significantly enhance the total Cronbach's *α*, and all items passed the discriminative power test. The Kaiser–Meyer–Olkin (KMO) value for factor analysis was 0.902, confirming the appropriateness of the data. Four items with communalities below 0.4 (Items 1, 10, 19, and 25) were excluded. The remaining 16 items clustered into three dimensions: interest, recognition/performance, and competence, with performance‐related items unexpectedly categorized within recognition.

#### 2.3.3 Confirmatory Factor Analysis

**FIGURE 1:**
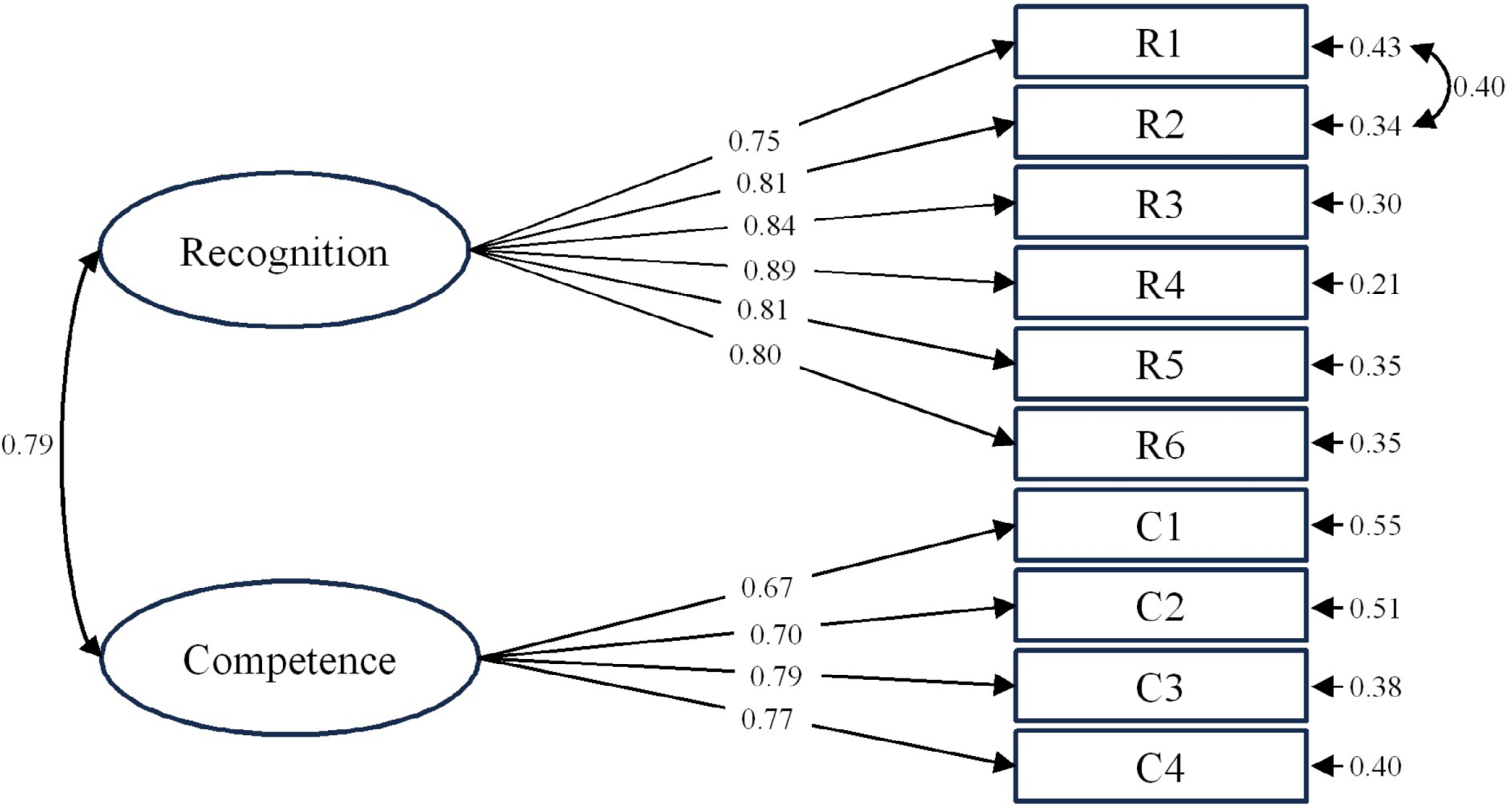
CFA model for the NIS standardized estimates. All the factor loadings are significant at the *p* < 0.001 level. “Recognition” and “Competence” are latent variables, and R1–C4 are observed variables. The standardized factor loadings between the latent variables and their respective observed variables indicate the explanatory power of each measurement indicator for the latent constructs. The bidirectional arrow represents the correlation between the latent variables (0.79). The measurement errors displayed to the right of the observed variables (e.g., 0.43, 0.34, etc.) represent the residual variances, or the portion of variance unexplained by the latent variables for each indicator. *N* = 416.

In the first‐order CFA, the Interest dimension had an average variance extracted (AVE) of 0.33, below the acceptable threshold of 0.5, and was therefore excluded. The model was refined by selecting modification indices (MI) greater than 10, leading to necessary modifications. The revised model, consisting of two dimensions and 10 items (Figure 1), demonstrated satisfactory fit indices: *χ* ^2^/df = 1.946, CFI = 0.978, RMSEA = 0.051. A subsequent second‐order CFA confirmed good fit indices (*χ* ^2^/df = 2.579, CFI = 0.967, RMSEA = 0.060), indicating that the two dimensions adequately represented “Naturalist identity.” We then compared the 10‐item, 2‐factor model with the 14‐item, 3‐factor (interest, recognition, competence) and 4‐factor models (interest, recognition, competence, performance). Model fit indices (AIC, BIC) showed that the 2‐factor structure provided the best fit: AIC_2‐factor_ = 6236, BIC_2‐factor_ = 6302, AIC_3‐factor_ = 8855, BIC_3‐factor_ = 8953, AIC_4‐factor_ = 11,098, BIC_4‐factor_ = 11,239.

### 2.4 Discussion

In Study 1, the development of the NIS involved a rigorous process of item generation, expert evaluation, and factor analysis to ensure its reliability and validity. The EFA identified three dimensions—interest, recognition/performance, and competence—although the Interest dimension was excluded in subsequent CFA due to inadequate AVE. The final scale, comprising two dimensions and 10 items, demonstrated good fit indices, supporting its stability and suitability for measuring naturalist identity. However, since the sample in Study 1 consisted of naturalists from fields such as birdwatching, botany, and entomology, we could not determine whether the psychometric properties of the NIS would be equally robust in the general population. Therefore, in Study 2, we conducted a cross‐group factor analysis between college students and naturalists to assess the usability of the NIS across different groups.

## 3 Study 2

### 3.1 Sample and Data Collection

We recruited a total of 442 college students (408 valid samples, see Table 2) from three academic disciplines—biology, economics, and power engineering—from a university in Yunnan province, who completed the final version of the NIS scale and a 2‐item naturalist identity scale used in a previous study (Meng et al. 2025). Demographic information for the sample in Study 2 is presented in Table 2.

**TABLE 2:** Demographic information of the sample in Study 2 (*N* = 408).

Variables	Number of participants	Percentage (%)
Gender		
Male	99	24.3
Female	309	75.7
Age		
< 18	13	3.2
18	186	45.6
19	107	26.2
20	66	16.2
> 20	36	8.8
Grade		
1	298	73.0
2	41	10.0
3	50	12.3
4	19	4.7
Major		
Economics	114	27.9
Power engineering	113	27.7
Biology	181	44.4

*Note:* Percentages may not total 100% exactly due to rounding.

### 3.2 Data Analysis

To assess measurement equivalence across different groups, we conducted a multiple‐group confirmatory factor analysis (MGCFA) framework, first establishing whether the measurement model adequately fit each group separately, followed by sequential tests of configural invariance (ensuring the same factor structure across groups), metric invariance (examining equality of factor loadings), and scalar invariance (assessing equality of intercepts) between the university student and naturalist groups (Fischer and Karl 2019). Model differences were interpreted based on changes in fit indices, with differences greater than 0.01 in CFI, 0.015 in RMSEA, and 0.030 (for metric invariance) or 0.015 (for scalar invariance) in standardized root mean square residual (SRMR) considered indicative of noninvariance, ensuring a rigorous assessment of whether the scale functioned equivalently across populations (Chen 2007). Furthermore, we compared the performance of NIS and the 2‐item scale among college students and naturalists. Data analysis was conducted using the R packages “psych” and “lavaan.”

### 3.3 Results

#### 3.3.1 Multiple‐Group Confirmatory Factor Analysis

Initially, the 2‐factor model showed an acceptable fit for both naturalists and college students, with CFI values above 0.95 and RMSEA values below 0.06. We then tested for configural invariance, which confirmed that the factor structure was consistent across both groups, with fit indices (CFI = 0.998, RMSEA = 0.023, SRMR = 0.027) meeting the criteria. Next, we examined factor loading invariance, assessing whether each item contributed equally to its respective factor across groups. The model passed this test with fit indices (CFI = 0.994, RMSEA = 0.037, SRMR = 0.039). Finally, we tested for scalar invariance to assess whether item intercepts were equivalent across groups. This test initially failed (CFI = 0.983, RMSEA = 0.054, SRMR = 0.058), indicating differences in intercepts. However, relaxing the intercept constraint on item R1 achieved scalar invariance (CFI = 0.986, RMSEA = 0.049, SRMR = 0.054). Overall, the results demonstrated minimal differences in the measurement structure between the two groups, supporting the cross‐group comparability of the scale. Subsequent analyses were conducted using the combined data for further descriptive statistics.

#### 3.3.2 Scale Psychometric Properties

The overall Cronbach's *α* for the newly developed scale was 0.925, with individual values for each dimension ranging from 0.817 to 0.920 (Table 3). All items had a CITC exceeding 0.5, and removing any item did not significantly improve Cronbach's *α*, indicating strong internal consistency. The CR for both dimensions was above the acceptable threshold of 0.6, further confirming the scale's reliability. To assess convergent validity, both AVE and CR were calculated. The AVE for the two factors exceeded 0.5, and CR exceeded 0.7, indicating satisfactory convergent validity. Discriminant validity was assessed using Pearson correlation coefficients and the square root of the AVE. The results showed that the square root of the AVE for each factor was greater than the maximum inter‐factor correlation, confirming good discriminant validity for the two dimensions.

**TABLE 3:** Measurement performance of the final version of the NIS (*n* = 824).

Scale items	Mean ± SD	CITC	Cronbach α	CR	AVE	Factor loading
Recognition subdimension	22.48 ± 4.82		0.920	0.922	0.664	
R1: I consider myself a naturalist.	3.81 ± 0.94	0.733				0.758
R2: My friends consider me a naturalist.	3.76 ± 0.93	0.780				0.816
R3: I constantly strive to become a more knowledgeable naturalist.	3.89 ± 0.93	0.767				0.854
R4: Being a naturalist has become an important part of my life.	3.77 ± 1.02	0.808				0.857
R5: I frequently use and browse naturalist‐related apps or websites.	3.50 ± 0.96	0.758				0.784
R6: I often share naturalist‐related knowledge and experiences with others.	3.75 ± 0.93	0.765				0.772
Competence subdimension	14.57 ± 2.73		0.817	0.824	0.541	
C1: I have acquired sufficient knowledge in my areas of interest within the natural world.	3.19 ± 0.98	0.600				0.669
C2: I can use reference materials to find the natural knowledge I need.	3.90 ± 0.75	0.596				0.701
C3: I can explain certain natural phenomena to others in a simple and understandable way.	3.73 ± 0.81	0.658				0.791
C4: When exploring or playing outdoors with friends, I often show sharper observational skills.	3.75 ± 0.84	0.680				0.774
Scale‐total	37.09 ± 7.01		0.925			

#### 3.3.3 Comparison Between the 2‐Item Scale With the NIS

**FIGURE 2:**
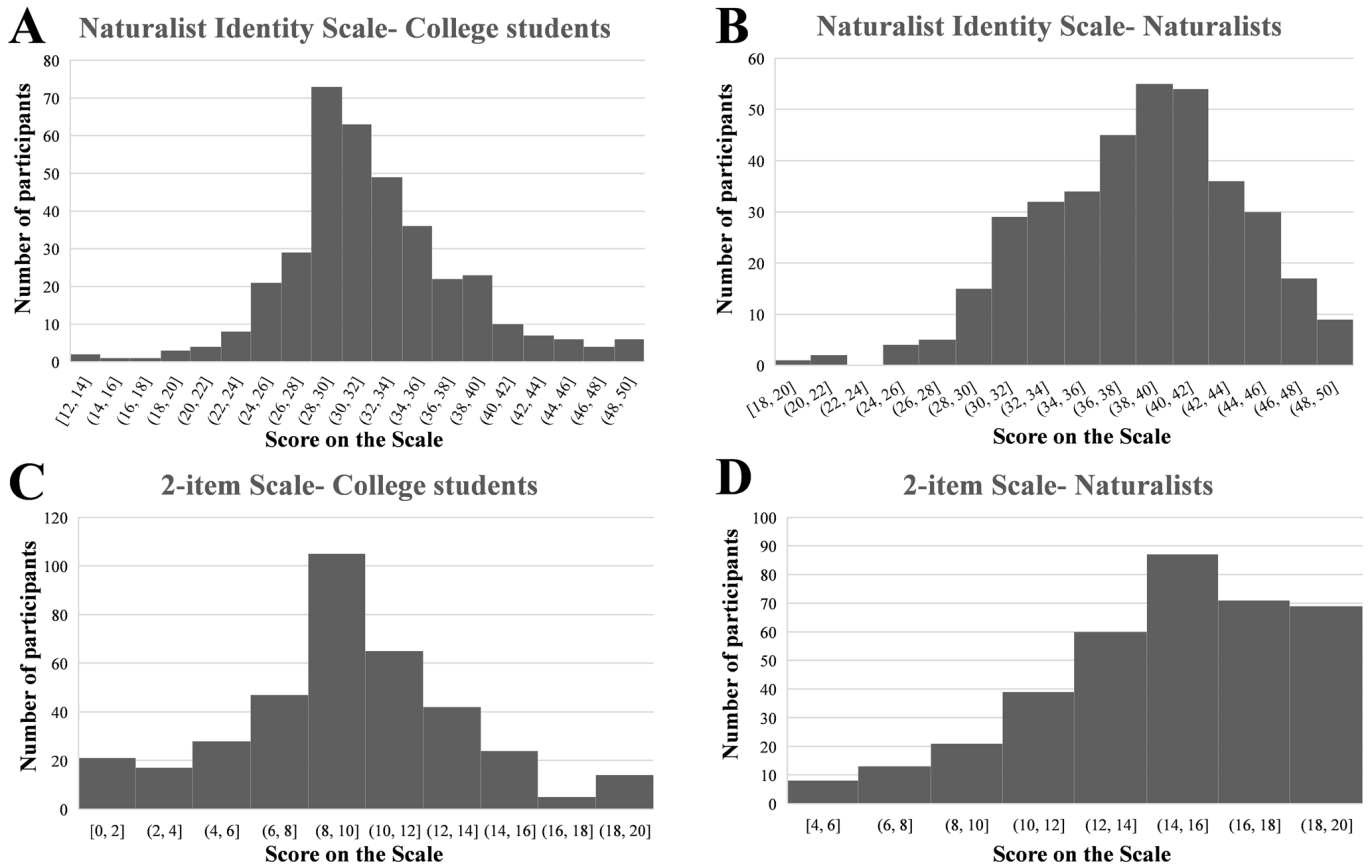
Score distribution of the 2‐item scale and naturalist identity scale among college students (*n* = 408) and naturalists (*n* = 416).

Pearson correlation analysis indicates that NIS and its two subdimensions are significantly correlated with the two single items of the 2‐item scale in both college students and naturalists (*p* < 0.001, see Table 4). Furthermore, to compare the measurement performance of the 2‐item scale and the NIS, we analyzed the score distributions for both scales among college students and naturalists. The results indicate that both scales exhibit a normal distribution among college students. In contrast, among naturalists, the 2‐item scale shows a ceiling effect, while the NIS scores continue to follow a normal distribution (Figure 2).

**TABLE 4:** The Pearson correlation of the 2‐item scale to the NIS.

	Naturalists		College students	
	Self‐identity	Identity from others	Self‐identity	Identity from others
Recognition	0.764***	0.706***	0.725***	0.716***
Competence	0.487***	0.463***	0.641***	0.627***
NIS	0.764***	0.737***	0.743***	0.731***

*** *p* < 0.001.

### 3.4 Discussion

In Study 2, the NIS was evaluated using MGCFA across college students and naturalists. The results showed that the factor structure was consistent across the two groups, although partial scalar invariance was observed, indicating some differences in item intercepts between the groups. The failure of full scalar invariance—resolved by freeing one intercept—suggests minor group‐specific differences in item interpretation. Compared with the 2‐item scale, the NIS showed superior measurement performance, particularly by avoiding ceiling effects among naturalists. These findings highlight the NIS's robustness in capturing naturalist identity across diverse populations and underscore its advantage over ultra‐short forms for nuanced assessment, offering a reliable tool for both academic research and practical applications in identity measurement.

## 4 Study 3

### 4.1 Sample

This study aims to validate the relationship between naturalist identity and biodiversity actions. Data for Study 3 were collected from two nature‐focused projects: the Naturalist Training Camp at the XTBG (April 2024) and the Weizhou Island Marine Observation Competition (March 2024), Guangxi province. Both projects attracted a substantial number of amateur naturalists. As in Study 1, participants in this study were recruited mainly from naturalist communities, whose members typically had some prior experience in nature observation and related knowledge; therefore, caution is warranted when generalizing the findings to the broader population. During the offline activities associated with these two projects, we explained the nature of our research to participants and invited them to participate in the study. Ultimately, we collected 206 questionnaires. After excluding 8 invalid questionnaires due to inconsistent responses to reverse‐coded items and excessively short response times, we had 198 valid questionnaires, resulting in an efficiency rate of 96.1%. Demographic information for the participants in this study is shown in Table 5.

**TABLE 5:** Demographic information of the sample in Study 3 (*N* = 198).

Items	Number of participants	Percentage (%)
Gender		
Male	94	47.5
Female	104	52.5
Age		
< 20	19	9.6
20–29	75	37.9
30–39	48	24.2
40–49	35	17.7
≥ 50	21	10.6
Education		
Middle school	6	3.0
High school	15	7.6
Undergraduate degree	114	57.6
Postgraduate degree	63	31.8
Income (RMB)		
< 3000	61	30.8
3001–5000	34	17.2
5001–10,000	52	26.3
10,001–20,000	31	15.7
> 20,000	20	10.1

*Note:* Percentages may not total 100% exactly due to rounding.

### 4.2 Data Collection

An electronic questionnaire was developed using Questionnaire Star. The instrument included the NIS, 2‐item identity scale, the Biodiversity Action Scale, the Sense of Obligation Scale, and demographic variables. The complete questionnaire comprised 26 items (Appendix D).

*Naturalist Identity*: The NIS, developed in Study 2, was used to assess naturalist identity. The scale consists of 10 items across two dimensions, including one item from the identity dimension: “I consider myself a naturalist.” Responses were scored using a five‐point Likert scale: where “1 = *Strongly Disagree*,” “2 = *Disagree*,” “3 = *Neutral*,” “4 = *Agree*,” “5 = *Strongly Agree*.” For comparative purposes, previously used items, “To what extent do you consider yourself a naturalist?” and “To what extent do others consider you a naturalist?” were also included (Meng et al. 2025). These single‐item questions were rated on a scale of 0–10.

*Biodiversity Actions*: To address the absence of suitable biodiversity conservation action scales for application in China, we adapted the Environmental Action Scale (Alisat and Riemer 2015; Li and Chen 2015) and the ProNature Behavior Scale (Barbett et al. 2020) to create a five‐item scale. An example item from the Biodiversity Action Scale is: “When I encounter incidents that harm wildlife, I actively intervene to stop them.” Responses were scored using a five‐point Likert scale, where “1 = *Never*,” “2 = *Rarely*,” “3 = *Sometimes*,” “4 = *Often*,” “5 = *Very Frequently*,” indicating frequency of behavior from 1 to 5.

*Sense of Obligation*: The Sense of Obligation for Biodiversity Scale was adapted from Powell et al. (2011) and Janmaimool and Khajohnmanee (2020) and consists of three items. One such item is: “I have a moral responsibility to protect biodiversity.” Responses were recorded on a five‐point Likert scale, where “1 = *Strongly Disagree*,” “2 = *Disagree*,” “3 = *Neutral*,” “4 = *Agree*,” “5 = *Strongly Agree*,” reflecting levels of agreement from 1 to 5.

### 4.3 Data Analysis

Descriptive statistics, including means and graphical summaries, were used to analyze demographic variables. The reliability of all scales was assessed using Cronbach's *α* coefficient. To examine the predictive power of various factors on biodiversity actions, we constructed four models and performed multiple regression analyses. Model 1 included only demographic variables as predictors. Model 2 and Model 3 separately tested the effects of naturalist identity and sense of obligation, respectively. Model 4 incorporated all variables simultaneously (see Table 6). To test the hypothesized mediation, we employed a mediation model with naturalist identity as the independent variable, biodiversity actions as the dependent variable, and sense of obligation as the mediator (testing H2 and H3). For the mediation model, standard errors and 95% confidence intervals for the parameter estimates were calculated using a nonparametric bootstrap method with 5000 samples, utilizing the “lavaan” package. Additionally, to explore potential bidirectional relationships, we tested an alternative model (with sense of obligation as the independent variable and naturalist identity as the mediator) and a reverse model (with biodiversity action as the independent variable and naturalist identity as the dependent variable). These additional models allowed us to examine the complex, dynamic interactions among the variables. Finally, Spearman correlation analysis was conducted to evaluate bivariate relationships. All data analyses and visualizations were performed using R (Version 4.2.0).

**TABLE 6:** Predicting biodiversity actions based on naturalist identity, sense of obligation, and demographic variables (*N* = 198).

	Model 1		Model 2		Model 3		Model 4	
	*b* (SE)	*t*	*b* (SE)	*t*	*b* (SE)	*t*	*b* (SE)	*t*
Age	0.134 (0.070)	1.917					0.147 (0.061)	2.427*
Income	0.058 (0.067)	0.873					0.063 (0.058)	1.09
Gender	0.088 (0.145)	0.603					0.212 (0.128)	1.66
Education	0.041 (0.127)	0.323					0.043 (0.110)	0.387
Naturalist identity			0.425 (0.064)	6.599**			0.362 (0.065)	5.548**
Sense of obligation					0.348 (0.067)	5.217**	0.256 (0.064)	4.002**
*F*	*F* (4,193) = 2.203		*F* (1,196) = 43.542		*F* (1,196) = 27.216		*F* (6,191) = 13.072	
*P*	0.070		0.000		0.000		0.000	
*R* ^2^	0.043		0.181		0.121		0.290	

*Note: b*, unstandardized regression coefficients, SE, standard error. In Gender, 1 = female, 0 = male.

* *p* < 0.05.

** *p* < 0.01.

### 4.4 Results

All scales in this study demonstrated strong internal consistency, with Cronbach's *α* values exceeding 0.7. Spearman correlation analysis revealed significant positive correlations among naturalist identity, biodiversity action, and sense of obligation (*p* < 0.01). Notably, male participants had higher naturalist identity scores than female participants. Age was positively correlated with biodiversity actions, while education level and income showed no significant correlations with naturalist identity, biodiversity action, or sense of obligation (Appendix C).

Multiple regression analysis was conducted to assess the impact of demographic variables, naturalist identity, and sense of obligation on biodiversity actions (Table 6). Model 1, which included only demographic variables, explained 4.3% of the variance (*R* ^2^ = 0.043). In Model 2, naturalist identity significantly predicted biodiversity actions (*b* = 0.425, *p* < 0.001, *R* ^2^ = 0.181). Model 3, which added sense of obligation, showed it as a significant predictor (*b* = 0.348, *p* < 0.001, *R* ^2^ = 0.121). The final model (Model 4), integrating all variables, accounted for 29.0% of the variance, with age, naturalist identity, and sense of obligation as significant predictors. Comparison with the 2‐item scale showed that the NIS explained an additional 2% of the variance in biodiversity conservation actions (Appendix E).

**FIGURE 3:**
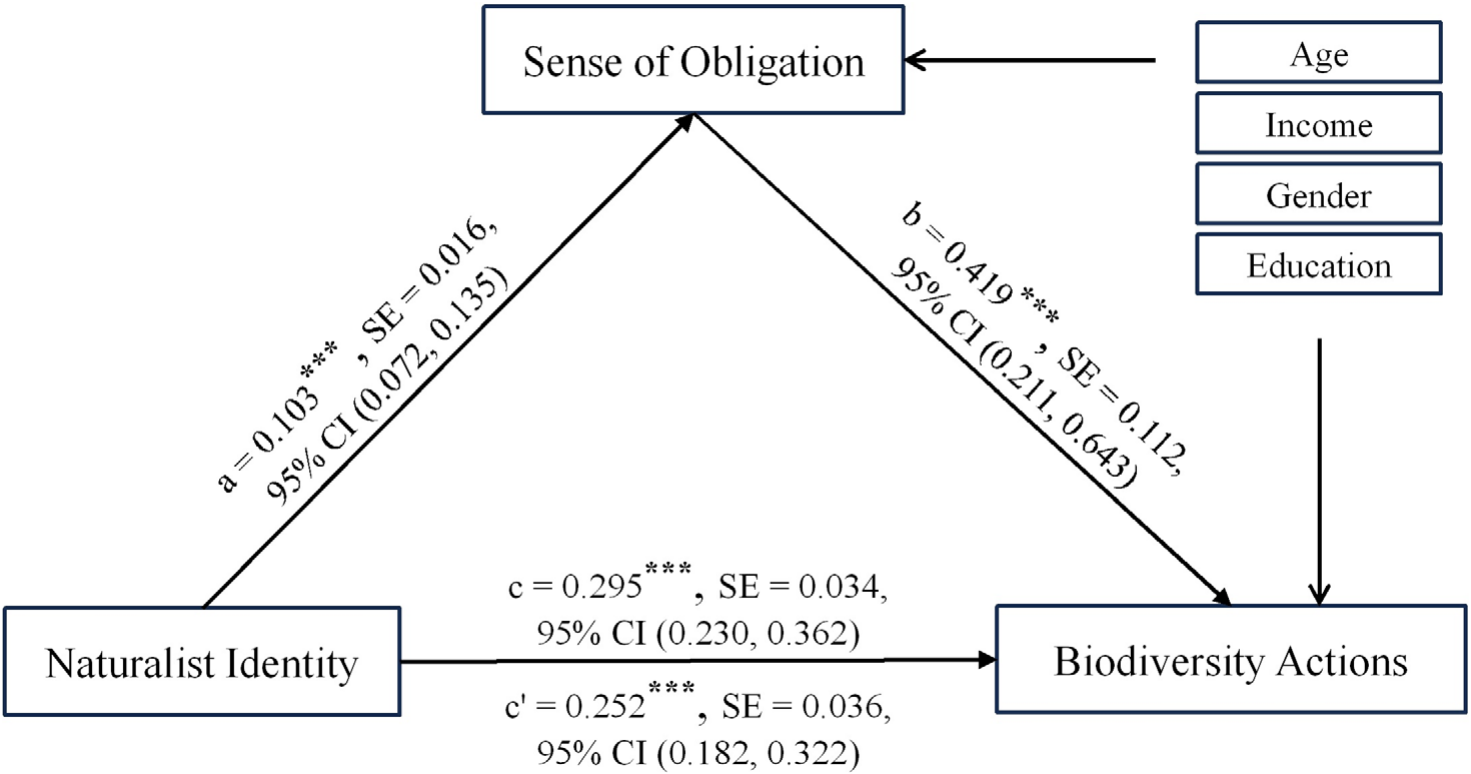
Diagram of intermediary analysis model. *N* = 198. ***p < 0.001. In the model, *c* denotes the total effect, and *c*′ denotes the direct effect. All path coefficients are unstandardized regression coefficients. Demographic variables (age, gender, income, and education) were controlled for in relation to the endogenous variable.

Mediation analysis revealed that a sense of obligation partially mediated the relationship between naturalist identity and biodiversity actions (Figure 3). The total effect was 0.295 (95% CI [0.230, 0.362], SE = 0.034, *p* < 0.001), which includes a mediation effect of 0.043 (95% CI [0.019, 0.076], SE = 0.015, *p* = 0.003) and a direct effect of 0.252 (95% CI [0.182, 0.322], SE = 0.036, *p* < 0.001). This model accounted for 28.2% of the variance in biodiversity actions. Additionally, we examined alternative and reverse mediation models. In the alternative model, the parameters were as follows: *a* = 0.946, *b* = 0.246, *c* = 0.663, *c*′ = 0.431. In the reverse model, the parameters were *a* = 0.167, *b* = 0.549, *c* = 0.691, and *c*′ = 0.599. In both models, the direct and indirect effects were statistically significant (*p* < 0.001).

To evaluate the potential issue of common method bias, we conducted Harman's single‐factor test. The first unrotated principal component explained 26.2% of the total variance, which is well below the commonly used thresholds of 40% and 50%. This result suggests that common method bias is unlikely to be a serious concern in the current dataset. Nevertheless, future research could further reduce this potential risk by incorporating multisource data (e.g., peer reports or behavioral observations) or adopting alternative research designs.

### 4.5 Discussion

In Study 3, the results indicate that naturalist identity is a significant predictor of biodiversity‐related actions, although the model accounts for a modest proportion of the variance (*R* ^2^ = 0.282). Naturalist identity has a direct effect on these actions and also influences them indirectly through a sense of obligation. Mediation analysis supports the hypothesis that a strong naturalist identity enhances a sense of moral responsibility, which subsequently drives actions aimed at biodiversity conservation. The NIS outperformed the two‐item scales, as it not only mitigated the ceiling effect but also accounted for additional variance in biodiversity actions, thereby illustrating its superior capacity to capture the complexity of naturalist identity. These findings emphasize the importance of cultivating naturalist identity to encourage active participation in biodiversity conservation efforts. However, the cross‐sectional nature of the study limits the ability to draw firm causal inferences. The significance of both the alternative and reverse models suggests that the relationships among naturalist identity, sense of obligation, and biodiversity actions may be more dynamic than a simple one‐way pathway. One possible interpretation is that engaging in biodiversity conservation behaviors may, in turn, strengthen individuals' naturalist identity and reinforce their sense of moral obligation, thereby forming a positive feedback loop. This process may occur even when initial participation is driven by external opportunities or situational demands, with repeated action gradually becoming integrated into one's identity and moral commitment. Although this interpretation remains preliminary, it highlights a promising direction for future longitudinal or experimental research to examine the reciprocal and potentially cyclical nature of these relationships.

## 5 General Discussion

This study developed the NIS, which demonstrates strong reliability and validity and is robust across diverse populations, including both naturalists and college students. Our findings indicate that naturalist identity is a significant predictor of biodiversity conservation actions, with a sense of obligation acting as a partial mediator. These results underscore the potential of conservation education to cultivate naturalist identity, thereby promoting actions aimed at biodiversity conservation.

While identity has been extensively explored in social psychology, research on naturalist identity and its influence on conservation actions remains limited. Existing measurement scales, such as the Environmental Identity Scale (Clayton 2003), primarily focus on environmental attitudes and norms, rather than personal or social identity (Walton and Jones 2018). This NIS represents a novel contribution by specifically addressing the identity of naturalists and is the first scale designed to quantify this aspect in a structured format. Our research demonstrates that naturalist identity comprises dimensions of recognition and competence, which align with the scientific identity model proposed by Carlone and Johnson's (2007). A notable feature of our model is the integration of recognition and performance into a single dimension within the NIS, indicating a strong correlation between naturalists' identities and their conservation behaviors. This relationship may be shaped by China's deep‐rooted cultural reverence for nature, particularly as reflected in Confucianism, Taoism, and Buddhism, which advocate for harmony with the natural environment (Li et al. 2015). In this context, identity is closely associated with observable, performance‐based actions that resonate with societal values of environmental stewardship (Wang et al. 2021).

It is important to note that the interest dimension was excluded from the final model due to its low AVE. Although model comparisons indicated that the two‐factor solution provided the best fit, this outcome may be partly influenced by the limitation of our sample composition in study 1, which primarily consisted of advanced naturalists. Given that interest plays a critical role in the early stages of identity development (Hazari et al. 2010; Hecht et al. 2019), future research involving novice or younger populations may more effectively capture the role of interest in the development of naturalist identity.

The second study extended the evaluation of the NIS by examining its applicability across different populations. Evidence of configural and metric invariance suggests that the construct of naturalist identity can be measured consistently among both naturalists and college students. However, the presence of only partial scalar invariance indicates subtle group‐specific differences in interpretation. This pattern is not uncommon in cross‐group identity research, where cultural or experiential contexts can influence item meaning (Chen 2007; Fischer and Karl 2019). Notably, the NIS outperformed the previously used 2‐item measure, which showed a ceiling effect among naturalists. This finding echoes broader concerns in environmental identity measurement, indicating that overly brief scales may compromise sensitivity and validity (Walton and Jones 2018).

In Study 3, we found that naturalist identity significantly predicted various biodiversity actions, supporting our hypothesis that a strong naturalist identity enhances environmental engagement. This result is consistent with identity theory, which asserts that behaviors are influenced by established identities (Oyserman et al. 2012; Stets and Burke 2000). Additionally, a sense of obligation emerged as a key predictor of biodiversity actions, aligning with the Norm Activation Model. This model suggests that when individuals perceive environmental threats as morally significant, they feel a sense of obligation to take action. Our findings support this theory, showing that naturalists' sense of obligation positively influences their conservation actions. More broadly, these results are consistent with the general mechanism proposed by identity theory and the Norm Activation Model, in which identity shapes behavior both directly and indirectly through moral obligation. However, this mechanism may manifest differently across specific cultural contexts. In the Chinese cultural context, deep‐rooted cultural principles, such as the Confucian ideal of “unity of heaven and humanity” (天人合一), Buddhist compassion for all living beings, and Taoist emphasis on harmonizing with nature, may further strengthen the link between identifying as a naturalist and feeling a personal obligation to protect biodiversity (Li and Wei 2023; Miller 2017). These traditions likely amplify the normative association between identity and obligation observed in our model. Therefore, while the overarching mechanism may be broadly consistent with universal psychological theories, its specific expression and strength may be culturally nuanced. Future cross‐cultural research is needed to further distinguish universal from culture‐specific aspects of this identity–obligation–action pathway.

Based on our findings, we propose that fostering naturalist identity should be incorporated into conservation education strategies. Policies can be more effective by promoting identity‐building approaches that support the development of naturalist identity from an early age. For instance, integrating local biodiversity into school curricula can stimulate students' interest in nature, creating opportunities for early identity development and long‐term commitment to conservation. Furthermore, policies should support community‐based conservation programs that emphasize recognition and competence, thereby fostering environmental stewardship as a cultural norm.

Educational programs can be designed to target the identity dimensions of recognition and competence identified in our research through structured activities. Citizen science projects, such as iNaturalist, provide an excellent platform for engaging individuals in biodiversity monitoring while enhancing their competence and recognition as contributors to scientific knowledge. By aligning these projects with identity‐enhancing mechanisms, such as developing skills in observation, species identification, information retrieval, and interpretation, along with fostering both self‐recognition and social recognition (e.g., celebrating participants' contributions to conservation efforts)—we can significantly strengthen participants' naturalist identity. These programs have the potential to cultivate long‐term commitment to biodiversity conservation by linking identity development with concrete environmental actions.

Despite the valuable insights gained from our study, several limitations should be acknowledged. First, all data were exclusively collected in China, which restricts the generalizability of the findings to other cultural contexts. For instance, the influence of a sense of obligation on behavior may vary significantly between Western and Eastern populations (Miller et al. 2011). Second, the samples in Studies 1 and 3 were primarily sourced from highly engaged communities (e.g., birdwatching groups, insect taxonomy networks) and participant pools of offline training programs. These individuals likely possessed stronger pre‐existing environmental motivations and behaviors, which may introduce sample homogeneity and self‐selection bias. Consequently, this could lead to an overestimation of the relationships between NIS, sense of obligation, and conservation actions, thereby limiting the generalizability of our findings to the broader public. Future research should explore the NIS and the mechanisms identified in this study across different age groups, including children, adolescents, and novice individuals. Third, while our mediation model indicated that naturalist identity predicts biodiversity‐related actions, it only explains a modest proportion of the variance, implying that additional factors may significantly influence these behaviors. Future work should incorporate other potential predictors, such as social norms, perceived behavioral control, or institutional support, to build a more comprehensive model. Fourth, the significance of both the alternative and reverse models suggests that the relationship among naturalist identity, sense of obligation, and conservation actions may be reciprocal rather than strictly unidirectional. In particular, conservation actions may not only result from identity and obligation, but may also reinforce them over time, forming a potentially cyclical process. Longitudinal or experimental designs are needed to clarify the directionality of these relationships. Finally, although the NIS has demonstrated reliability and validity in cross‐sectional surveys, its applicability in longitudinal intervention studies has yet to be established. Prospective studies tracking identity development and behavioral change over time are recommended to further validate the scale and its predictive utility.

## Funding

This work was supported by Xishuangbanna Tropical Botanical Garden, Chinese Academy of Sciences (E2ZK312B01).

## Ethics Statement

This study was conducted in accordance with the ethical guidelines established by XTBG and received ethical approval from the Biomedical Ethics Committee of XTBG (Reference No: XTBG‐2024‐008). Prior to the initiation of the study, participants were provided with comprehensive information regarding the research objectives, methodologies, benefits, and potential risks, communicated through both written and verbal formats. Participants were told that submitting the results of the online questionnaire meant agreeing to participate in the study and that they had the right to withdraw from the study during the filling out process. The confidentiality of participant identities was strictly maintained, and their information was used solely for analytical purposes.

## Conflicts of Interest

The authors declare no conflicts of interest.

## Supporting information


**Appendix A.** Interview outline and interviewees' information.
**Appendix B**. Questionnaire used in the Study 1 (translated from Chinese).
**Appendix C**. The original 25‐item pool and the 20 items retained for factor analysis.
**Appendix D**. Questionnaire used in the Study 3 (translated from Chinese).
**Appendix E**. Correlation matrix and regression models using the 2‐item naturalist identity scale in Study 3.
**Table E1**. Spearman correlation for each variable in Study 3 (*N* = 198).
**Table E2**. Predicting biodiversity actions based on naturalist identity (2‐item scale), obligation and demographic variables (*N* = 198).

## Data Availability

The data that support the finding of this study are available on request from the corresponding author. The data are not publicly available due to privacy or ethical restrictions.
