# Supplementary material for: Naturalist Identity and Biodiversity Conservation: The Mediating Role of Obligation
Source: Psych J. 2026 May 4;15(3):e70098. doi: 10.1002/pchj.70098 (PMC13137115; doi:10.1002/pchj.70098)
Supplement: Supplementary file 1 — Appendix A. Interview outline and interviewees' information. Appendix B. Questionnaire used in the Study 1 (translated from Chinese). Appendix C. The original 25‐item pool and the 20 items retained for factor analysis. Appendix D. Questionnaire used in the Study 3 (translated from Chinese). Appendix E. Correlation matrix and regression models using the 2‐item naturalist identity scale in Study 3. Table E1. Spearman correlation for each variable in Study 3 (N = 198). Table E2. Predicting biodiversity actions based on naturalist identity (2‐item scale), obligation and demographic variables (N = 198). [file PCHJ-15-e70098-s001.docx]

**Appendix A. Interview outline and interviewees’ information**

**I. Interview outline (translated from Chinese)**

1. Please provide a brief introduction of yourself.
2. Do you consider yourself a naturalist? If so, please explain your reasoning.
3. Do others perceive you as a naturalist? If yes, please elaborate on their reasoning.
4. How does your identity as a naturalist influence your work, personal life, and social interactions?
5. As a naturalist, what specific areas of interest do you pursue?
6. What do you believe are your competencies as a naturalist?
7. What behaviors do you demonstrate that align with your identity as a naturalist?
8. In addition to your identity as a naturalist--encompassing recognition, performance, competence, and interests—are there any other aspects you would like to discuss?

**II. Some information about the interviewee (n = 10)**

No. 1: **Gender**: female; **Age**: 27; **Self-Reported Identity Score**: 10; **Years of Participation in Nature Observation/Activities**: 6; **Occupation**: research scientist, environmental artist, serious amateur naturalist; **Specific Interests on Nature**: plants, reptiles, amphibians, birds, insects, fungi, clouds.

No. 2: **Gender**: male; **Age**: 26; **Self-Reported Identity Score**: 7; **Years of Participation in Nature Observation/Activities**: 5; **Occupation**: environmental educator, serious amateur naturalist; **Specific Interests on Nature**: insects (mantises, orthopteran, phasmids), plants.

No. 3: **Gender**: male; **Age**: 23; **Self-Reported Identity Score**: 10; **Years of Participation in Nature Observation/Activities**: 11; **Occupation**: research scientist, popular science personnel; **Specific Interests on Nature**: myriapods, insects, spiders.

No. 4: **Gender**: male; **Age**: 28; **Self-Reported Identity Score**: 10; **Years of Participation in Nature Observation/Activities**: 5; **Occupation**: environmental educator, serious amateur naturalist, horticulturist; **Specific Interests on Nature**: plants, insects.

No. 5: **Gender**: male; **Age**: 39; **Self-Reported Identity Score**: 10; **Years of Participation in Nature Observation/Activities**: 19; **Occupation**: research scientist, horticulturist; **Specific Interests on Nature**: plants (Fabaceae, Magnoliaceae, Rosaceae, Asteraceae).

No. 6: **Gender**: male; **Age**: 30; **Self-Reported Identity Score**: 7; **Years of Participation in Nature Observation/Activities**: 9; **Occupation**: nature conservationist, serious amateur naturalist; **Specific Interests on Nature**: astronomy, birds, spiders, insects, mammals, reptiles, amphibians.

No. 7: **Gender**: male; **Age**: 42; **Self-Reported Identity Score**: 10; **Years of Participation in Nature Observation/Activities**: 23; **Occupation**: environmental artist, environmental educator, horticulturist, popular science personnel; **Specific Interests on Nature**: plants (flowering plants), insects (aesthetically appealing insects).

No. 8: **Gender**: male; **Age**: 44; **Self-Reported Identity Score**: 9; **Years of Participation in Nature Observation/Activities**: 20; **Occupation**: research scientist, popular science personnel; **Specific Interests on Nature**: minerals, geological phenomena, stones, mountains.

No. 9: **Gender**: male; **Age**: 40; **Self-Reported Identity Score**: 9; **Years of Participation in Nature Observation/Activities**: 17; **Occupation**: research scientist; **Specific Interests on Nature**: plants, insects.

No. 10: **Gender**: male; **Age**: 41; **Self-Reported Identity Score**: 6; **Years of Participation in Nature Observation/Activities**: 12; **Occupation**: nature conservationist, environmental educator, serious amateur naturalist; **Specific Interests on Nature**: plants, insects, astronomy.

**Appendix B. Questionnaire used in the Study 1 (translated from Chinese)**

Dear Naturalists

Thank you very much for participating in this survey. The purpose of this survey is to study the structure and composition of naturalist identity. Your insights will provide us with valuable information. This survey is anonymous and consists of 25 items, taking approximately 5 minutes to complete. There are no right or wrong answers, so please respond objectively. We assure you that all data will be kept strictly confidential and will only be used for research analysis. Your participation is entirely voluntary. Once you have completed the survey, please submit it. Completing and submitting the results of the online questionnaire means you agree to participate in the study, and you have the right to withdraw from the study during the completion process. Thank you for your contribution to biodiversity conservation research!

Note: "Naturalists" refers to individuals who, driven by an inherent passion and love, actively study and explore various aspects of natural objects such as animals, plants, and ecosystems.

Age: ______

Gender:

□Male

□Female

Education:

□Middle school or below

□High school

□Undergraduate degree

□Postgraduate degree

Please choose your opinion on the following statements according to your real opinion. The options are divided into strongly disagree, disagree, not sure, agree, strongly agree.

1. I have a strong curiosity about the nature around me.

□strongly disagree □disagree □not sure □agree □strongly agree

1. Being in nature makes me very happy.

□strongly disagree □disagree □not sure □agree □strongly agree

1. I am interested in learning about most aspects of the natural world.

□strongly disagree □disagree □not sure □agree □strongly agree

1. I am particularly passionate about learning one specific category of nature, such as animals, plants, or minerals.

□strongly disagree □disagree □not sure □agree □strongly agree

1. I consider myself a naturalist.

□strongly disagree □disagree □not sure □agree □strongly agree

1. My friends consider me a naturalist.

□strongly disagree □disagree □not sure □agree □strongly agree

1. I constantly strive to become a more knowledgeable naturalist.

□strongly disagree □disagree □not sure □agree □strongly agree

1. Being a naturalist has become an important part of my life.

□strongly disagree □disagree □not sure □agree □strongly agree

1. When I encounter unfamiliar naturalist-related questions, I can always find relevant information or seek help from friends.

□strongly disagree □disagree □not sure □agree □strongly agree

1. I have acquired sufficient knowledge in my areas of interest within the natural world.

□strongly disagree □disagree □not sure □agree □strongly agree

1. I can use reference materials to find the natural knowledge I need.

□strongly disagree □disagree □not sure □agree □strongly agree

1. I can explain certain natural phenomena to others in a simple and understandable way.

□strongly disagree □disagree □not sure □agree □strongly agree

1. When exploring or playing outdoors with friends, I often show sharper observational skills.

□strongly disagree □disagree □not sure □agree □strongly agree

1. Others frequently ask me for advice on naturalist topics.

□strongly disagree □disagree □not sure □agree □strongly agree

1. I have invested a lot of time or money in my passion for nature.

□strongly disagree □disagree □not sure □agree □strongly agree

1. I often record natural objects through photography or other means.

□strongly disagree □disagree □not sure □agree □strongly agree

1. I frequently use and browse naturalist-related apps or websites.

□strongly disagree □disagree □not sure □agree □strongly agree

1. I often share naturalist-related knowledge and experiences with others.

□strongly disagree □disagree □not sure □agree □strongly agree

1. I have many friends who are also naturalists.

□strongly disagree □disagree □not sure □agree □strongly agree

1. I often read books or watch documentaries about nature.

□strongly disagree □disagree □not sure □agree □strongly agree

Please choose your opinion on the following statements according to your real opinion. Responses ranged from 0 "Not at all" to 10 "Completely".

1. To what extent do you consider yourself a naturalist?

□ 0 □ 1 □ 2 □ 3 □ 4 □ 5 □ 6 □ 7 □ 8 □ 9 □ 10

1. To what extent do others consider you a naturalist?

□ 0 □ 1 □ 2 □ 3 □ 4 □ 5 □ 6 □ 7 □ 8 □ 9 □ 10

**Appendix C. The original 25-item pool and the 20 items retained for factor analysis**

| NO. | Original scale items | Sub-dimensions | Source | Number of participants mentioned |
| --- | --- | --- | --- | --- |
| 1 | I have a strong curiosity about the nature around me. | Interest | Adapted from Hazari et al., 2010 |  |
| 2 | Being in nature makes me very happy. | Interest | Interview | 5 |
| 3 | I am interested in learning about most aspects of the natural world. | Interest | Interview | 3 |
| 4 | I am particularly passionate about learning one specific category of nature, such as animals, plants, or minerals. | Interest | Interview | 5 |
| 5 | I am willing to learn more about nature through various channels. (removed in expert review) | Interest | Adapted from Hosbein & Barbera, 2020 |  |
| 6 | *I consider myself a naturalist. | Recognition | Interview; Adapted from Walton & Jones, 2018 | 5 |
| 7 | * My friends consider me a naturalist. | Recognition | Interview; Adapted from Walton & Jones, 2018 | 8 |
| 8 | *I constantly strive to become a more knowledgeable naturalist. | Recognition | Interview | 2 |
| 9 | *Being a naturalist has become an important part of my life. | Recognition | Adapted from Walton & Jones, 2018 |  |
| 10 | When I encounter unfamiliar naturalist-related questions, I can always find relevant information or seek help from friends. | Competence | Interview | 4 |
| 11 | *I have acquired sufficient knowledge in my areas of interest within the natural world. | Competence | Interview | 4 |
| 12 | I believe that through my efforts, I can understand complex natural knowledge and phenomena. (removed in expert review) | Competence | Adapted from Hazari et al., 2010 |  |
| 13 | *I can use reference materials to find the natural knowledge I need. | Competence | Interview | 4 |
| 14 | *I can explain certain natural phenomena to others in a simple and understandable way. | Competence | Interview | 5 |
| 15 | *When exploring or playing outdoors with friends, I often show sharper observational skills. | Competence | Interview | 5 |
| 16 | I have strong inductive ability when observing nature. (removed in expert review) | Competence | Interview | 3 |
| 17 | Others frequently ask me for advice on naturalist topics. | Competence | Interview | 5 |
| 18 | I often collect elements from nature. (removed in expert review) | Performance | Interview | 2 |
| 19 | I have invested a lot of time or money in my passion for nature. | Performance | Adapted from Cheung et al., 2016 |  |
| 20 | I often record natural objects through photography or other means. | Performance | Interview | 4 |
| 21 | I am used to observing the nature around me. (removed in expert review) | Performance | Interview | 3 |
| 22 | *I frequently use and browse naturalist-related apps or websites. | Performance | Interview | 2 |
| 23 | *I often share naturalist-related knowledge and experiences with others. | Performance | Interview | 8 |
| 24 | I have many friends who are also naturalists. | Performance | Interview | 5 |
| 25 | I often read books or watch documentaries about nature. | Performance | Interview | 8 |

Note: *N* indicates the number of participants who mentioned the item.

All items were surveyed in Chinese, and the English version in the table was translated by two doctoral students majoring in environmental education.

* The final items for Naturalist Identity Scale

**Appendix D. Questionnaire used in the Study 3 (translated from Chinese)**

Dear Naturalists,

Thank you very much for participating in this survey. The purpose of this survey is to better understand how the identity of naturalists influences biodiversity conservation behaviors. Your insights will provide us with valuable information. This survey is anonymous and consists of 24 items, taking approximately 5 minutes to complete. There are no right or wrong answers, so please respond objectively. We assure you that all data will be kept strictly confidential and will only be used for research analysis. Your participation is entirely voluntary. Once you have completed the survey, please submit it. Completing and submitting the results of the online questionnaire means you agree to participate in the study, and you have the right to withdraw from the study during the completion process. Thank you for your contribution to biodiversity conservation research!

Note: "Naturalists" refers to individuals who, driven by an inherent passion and love, actively study and explore various aspects of natural objects such as animals, plants, and ecosystems.

Age (年龄): ______

Gender (性别):

□Male

□Female

Education (受教育程度):

□Junior middle education or below

□Senior middle education

□Undergraduate college

□Postgraduate or above

Income (RMB/month) (收入，元/月):

□0-3000

□3001-5000

□5001-10000

□10001-20000

□＞20000

Please choose your opinion on the following statements according to your real opinion. The options are divided into strongly disagree, disagree, not sure, agree, strongly agree.

(1) I consider myself a naturalist (我认为我是一名博物爱好者).

□strongly disagree □disagree □not sure □agree □strongly agree

(2) My friends consider me a naturalist (我的朋友认为我是一名博物爱好者).

□strongly disagree □disagree □not sure □agree □strongly agree

(3) I constantly strive to become a more knowledgeable naturalist (我一直努力使得自己成为更加知识渊博的博物爱好者).

□strongly disagree □disagree □not sure □agree □strongly agree

(4) Being a naturalist has become an important part of my life (博物爱好已经成为我生活的重要组成部分).

□strongly disagree □disagree □not sure □agree □strongly agree

(5) I frequently use and browse naturalist-related apps or websites (我经常使用和浏览博物相关的APP或网站).

□strongly disagree □disagree □not sure □agree □strongly agree

(6) I often share naturalist-related knowledge and experiences with others (我经常与他人交流博物相关的知识和经验).

□strongly disagree □disagree □not sure □agree □strongly agree

(7) I have acquired sufficient knowledge in my areas of interest within the natural world (我对感兴趣的自然领域已经掌握足够多的相关知识).

□strongly disagree □disagree □not sure □agree □strongly agree

(8) I can use reference materials to find the natural knowledge I need (我能够借助资料工具，查找到所需的自然知识).

□strongly disagree □disagree □not sure □agree □strongly agree

(9) I can explain certain natural phenomena to others in a simple and understandable way (我能够用通俗易懂的语言向别人解释自然界中的一些现象).

□strongly disagree □disagree □not sure □agree □strongly agree

(10) When exploring or playing outdoors with friends, I often show sharper observational skills (在和朋友一起在野外考察或者游玩，我往往表现出更加敏锐的观察力).

□strongly disagree □disagree □not sure □agree □strongly agree

Please choose your opinion on the following statements according to your real opinion. The options are divided into strongly disagree, disagree, not sure, agree, strongly agree.

1. I have an ethical responsibility to protect biodiversity (我有保护生物多样性的道德责任).

□strongly disagree □disagree □not sure □agree □strongly agree

1. I feel obligated to persuade those around me to protect wildlife as much as possible (我觉得我有义务劝说周围的人尽量保护野生动植物).

□strongly disagree □disagree □not sure □agree □strongly agree

1. I feel capable of contributing to biodiversity conservation. (我觉得我有能力为生物多样性保护做贡献).

□strongly disagree □disagree □not sure □agree □strongly agree

Please choose your opinion on the following statements according to your real opinion. Responses ranged from 0 "Not at all" to 10 "Completely".

1. To what extent do you consider yourself a naturalist (我多大程度上认为我是一名博物爱好者)?

□ 0 □ 1 □ 2 □ 3 □ 4 □ 5 □ 6 □ 7 □ 8 □ 9 □ 10

1. To what extent do others consider you a naturalist (我的朋友多大程度上认为我是一名博物爱好者)?

□ 0 □ 1 □ 2 □ 3 □ 4 □ 5 □ 6 □ 7 □ 8 □ 9 □ 10

Please select an option based on your frequency of behavior in the following statements. The options are divided into never, rarely, sometimes, often, and very frequently.

1. I often discuss biodiversity conservation issues with colleagues or friends (我和同事或者朋友讨论有关生物多样性保护的问题).

□never □rarely □sometimes □often □very frequently

1. When I encounter events that harm wildlife or plants, I proactively intervene to discourage it (我遇到破坏野生动植物的事件，主动上前予以劝阻).

□never □rarely □sometimes □often □very frequently

1. I have reported incidents of wildlife or plant destruction to the authorities (我曾向执法部门举报破坏野生动植物的事件).

□never □rarely □sometimes □often □very frequently

1. I have donated money to biodiversity protection organizations (我曾向生物多样性保护组织捐钱).

□never □rarely □sometimes □often □very frequently

1. I have participated in public welfare activities related to biodiversity conservation (我参加过和生物多样性保护相关的公益活动).

□never □rarely □sometimes □often □very frequently

**Appendix E. Correlation matrix and regression models using the 2-item naturalist identity scale in Study 3**

**Table E1. Spearman correlation for each variable in study 3 (N = 198)**

|  | Identity | Sense of obligation | Biodiversity action | Age |
| --- | --- | --- | --- | --- |
| Identity | 1 |  |  |  |
| Sense of obligation | 0.327** | 1 |  |  |
| Biodiversity action | 0.460** | 0.366** | 1 |  |
| Age | 0.024 | -0.083 | 0.172* | 1 |

Note: *p < 0.05, **p < 0.01.

In Gender, 1 = female, 0 = male.

**Table E2. Predicting biodiversity actions based on naturalist identity (2-item scale), obligation and demographic variables (N = 198)**

|  | Model 1 | | Model 2 | | Model 3 | | Model 4 | |
| --- | --- | --- | --- | --- | --- | --- | --- | --- |
|  | b (SE) | t | b (SE) | t | b (SE) | t | b (SE) | t |
| Age | 0.134  (0.070) | 1.917 |  |  |  |  | 0.604  (0.214) | 2.823* |
| Income | 0.058  (0.067) | 0.873 |  |  |  |  | 0.188  (0.204) | 0.918 |
| Gender | 0.088  (0.145) | 0.603 |  |  |  |  | 0.628  (0.447) | 1.406 |
| Education | 0.041  (0.127) | 0.323 |  |  |  |  | -0.061  (0.389) | -0.157 |
| Naturalist identity |  |  | 0.377  (0.062) | 6.117** |  |  | 0.324  (0.063) | 5.082** |
| Sense of obligation |  |  |  |  | 0.348  (0.067) | 5.217** | 0.538  (0.120) | 4.479** |
| F | F (4,193) = 2.203 | | F (1,196) = 47.422 | | F (1,196) = 27.216 | | F (6,191) = 12.739 | |
| P | 0.070 | | 0.000 | | 0.000 | | 0.000 | |
| R^2^ | 0.043 | | 0.160 | | 0.121 | | 0.261 | |

Note: * *p* < 0.05, ** *p* < 0.01. *b* unstandardized coefficients, *SE* standard error. In Gender, 1 = female, 0 = male.
